# The influence of dental experience on a dental implant navigation system

**DOI:** 10.1186/s12903-019-0914-2

**Published:** 2019-10-17

**Authors:** Ting-Mao Sun, Huey-Er Lee, Ting-Hsun Lan

**Affiliations:** 1 School of Dentistry, College of Dental Medicine, Kaohsiung Medical University, 100 Shin-Chuan 1st Road, Sanmin District, Kaohsiung, 80708 Taiwan; 2 Division of Family Dentistry, Department of Dentistry, Kaohsiung Medical University Hospital, Kaohsiung Medical University, Kaohsiung, Taiwan; 3 Division of Prosthodontics, Department of Dentistry, Kaohsiung Medical University Hospital, Kaohsiung Medical University, Kaohsiung, Taiwan

**Keywords:** Accuracy, Dental implant, Navigation system, Surgical experience

## Abstract

**Background:**

This study evaluated the operating performance of an implant navigation system used by dental students and dentists of prosthodontic background with varying levels of experience. A surgical navigation system and optical tracking system were used, and dentists’ accuracies were evaluated in terms of differences between the positions of actually drilled holes and those of the holes planned using software before surgeries.

**Methods:**

The study participants were 5 dental students or dentists who had studied in the same university and hospital but had different experience levels regarding implants. All participants were trained in operating the AqNavi system in the beginning of the study. Subsequently, using 5 pairs of dental models, each participant drilled 5 implant holes at 6 partially edentulous positions (11, 17, 26, 31, 36, and 47). In total, each participant conducted 30 drilling tests.

**Results:**

In total, 150 tests among 5 dentists at 6 tooth positions (11, 17, 26, 31, 36, and 47) were conducted. Although a comparison of the tests revealed significant differences in the longitudinal error (*P* < .0001) and angular error (*P* = .0011), no significant difference was observed in the total error among the dentists.

**Conclusions:**

A relatively long operating time was associated with relatively little implant experience. Through the dental navigation system, dental students can be introduced to dental implant surgery earlier than what was possible in the past. The results demonstrated that the operational accuracy of the dental implant navigation system is not restricted by participants’ implant experience levels. The implant navigation system assists the dentist in the ability to accurately insert the dental implant into the correct position without being affected by his/her own experience of implant surgery.

## Background

Prosthetic-driven surgical placement of dental implants is crucial in implant dentistry, and it facilitates harmonization with surrounding gingival tissues, the alveolar process, and adjacent teeth to restore patient’s appearance and function. To achieve the aforementioned goals, it is crucial to control the relative positions, angles, and directions of artificial implants. A surgical guide made using CAD-CAM (Computer aided design/manufacturing) or a real-time navigation system can help a clinician control and reduce risks. Thus, a comprehensive treatment plan that considers unique anatomical structures, such as the maxillary sinus and inferior alveolar nerve, can be used to determine the position and depth of inserting the implant to increase the chances of success, thereby reducing the likelihood of sequelae [1, 2].

A dental implant navigation system integrates implanting instruments, medical imaging, optical positioning devices, and preoperational implant planning software to realize preoperational clinical planning in real time and to guide the operator to drill at the planned position [3]. Birkfellner et al. [4] argued that such a guidance system can reduce the intraoperative risk of damage to critical anatomic structures. Sießegger et al. [5] proved that the image-guided navigation system is a valuable tool in implant dentistry and guided system techniques are superior to conventional implant techniques. Watzinger et al. [6] used the surgical navigation to place implants over the zygoma areas of cadavers, with a success rate of 80%; they also suggested that an improved visualization technique might improve the accuracy of the procedure. To summarize the aforementioned results, numerous dentists are convinced of the potential advantages of navigation systems; however, the accuracy and reliability of any navigation system depend on dentists’ familiarity with the hardware and software [7].

The concept of learning curves was first used in medical care in the 1970s; Nowitzke et al. [8] reported that this concept has been widely discussed for minimal access surgery. In learning curves, Waldman et al. [9] demonstrated that 3 major theories are applied: 1) repetition reduces the unit production time; 2) the unit production time decreases over time; and 3) adhering to predictable modes decreases the unit production time. Repeated operation of a dental implant guidance system reduces the operating time, and maximum precision can be attained over time. Sun et al. [10] constructed a learning curve model for operating an implant guidance system, and they indicated that their system had to be operated at least 5 times to ensure the safety and reliability of the surgery. On the other hand, Breaux et al. [11] reported that surgeons with advanced experience show significantly lower operating time, but morbidity and mortality remain low even though plateau of learning curve had not been reached. Block et al. [12] showed that different dentists had similar implant installation accuracies after their learning curve plateaus were reached.

To learn a new skill or equipment, the influence of clinician’s experience was still an interesting topic. The purpose of this study is to compare the operating performance of a dental navigation system by dental prosthodontics students and dentists with varying levels of experience under reaching the learning platform. Moreover, becoming an expert in the implantation field of whether the navigation could shorten the dentist’s dental implant training schedule and lower the threshold for entry into this field. This study hypothesis that the dentists with different experience by using dental navigation system without difference in accuracy.

## Methods

This study has been approved by the Human Research Ethics Committee of the Kaohsiung Medical University Hospital (IRB code: KMUH-IRB-2013-08-02(1)). Informed assent/consent was obtained for five dentists prior to any research activities. This is a series of research designs related to the accuracy of dental navigation system by different study methods.

### Main system

**Fig. 1:**
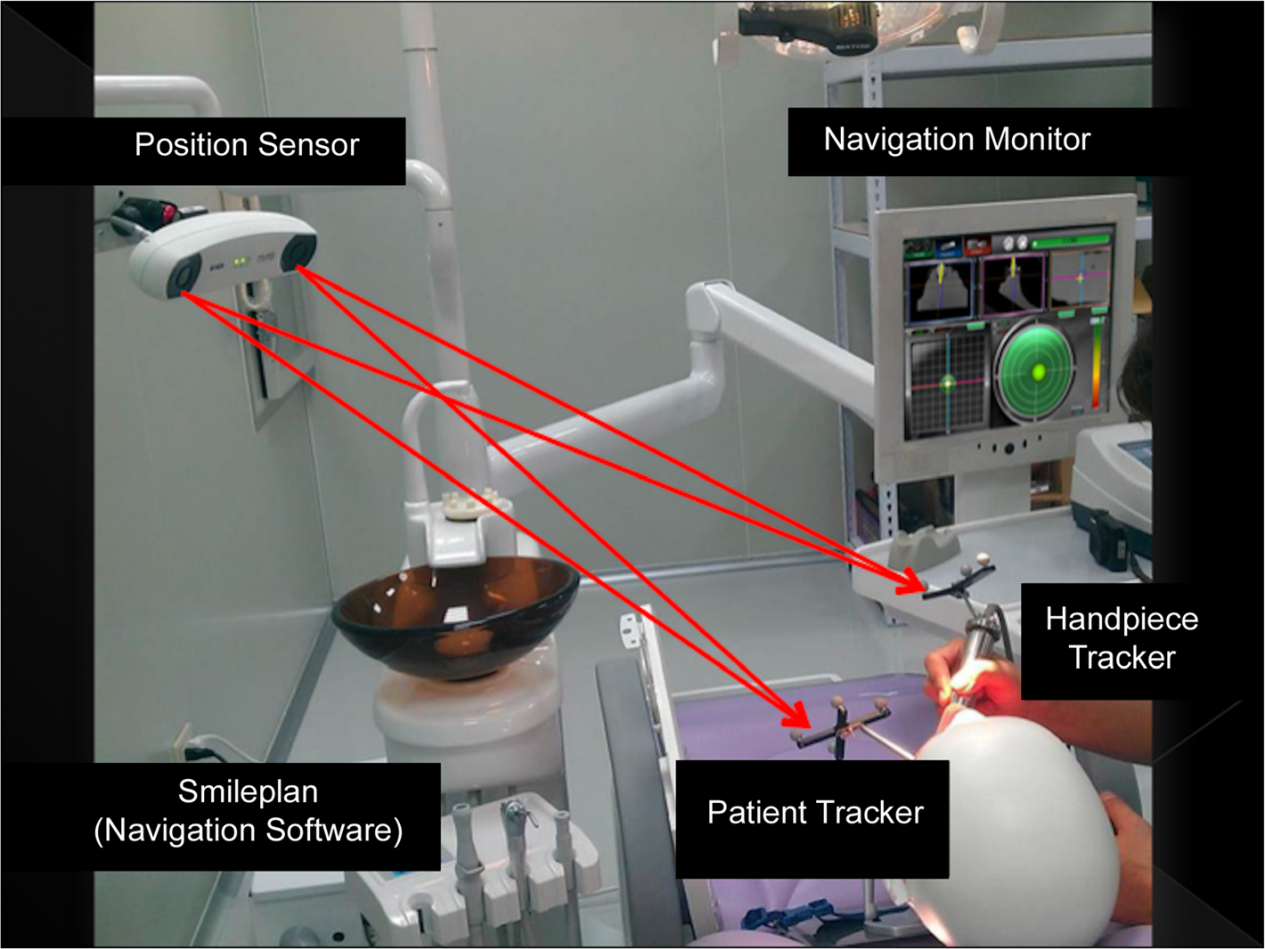
This dental implant navigation system is composed of 3 parts. Cone beam computed tomography (CBCT) and SmilePlan are used to set up the implant treatment plan. A position sensor tracks the handpiece and the patient’s movements in real time. AqNavi monitor guides the surgeon in positioning the handpiece in the optimal location for accurate drilling. (Figure was provided by Taiwan Implant Technology Company, Ltd.)

This study employed the Aq Navi Surgical Navigation System (AqNavi System) manufactured by Taiwan Implant Technology Company, Ltd. and the Polaris Vicra optical tracking system developed by Northern Digital Inc. (Fig. 1).

### Main operator

The study participants were 5 dental students or dentists who had studied at the same university (Kaohsiung Medical University, Taiwan, ROC) and hospital (Kaohsiung Medical University Hospital, Taiwan, ROC) but had different implant experience levels and had never used a navigation system before. Gladwell [13] lay out the different experience levels through scientific data analysis and philosophical demonstration were classified as follows:

1. Visiting staff (VS) who had at least 10,000 h of clinical training.
2. Chief resident (CR) who had 8000 h of clinical training.
3. Resident (R) who had 6000 h of clinical training.
4. Dental intern (DI) who had 3000 h of clinical training.
5. Dental clerk (DC) who had 1000 h of clinical training.

### Training before drilling test

**Fig. 2:**
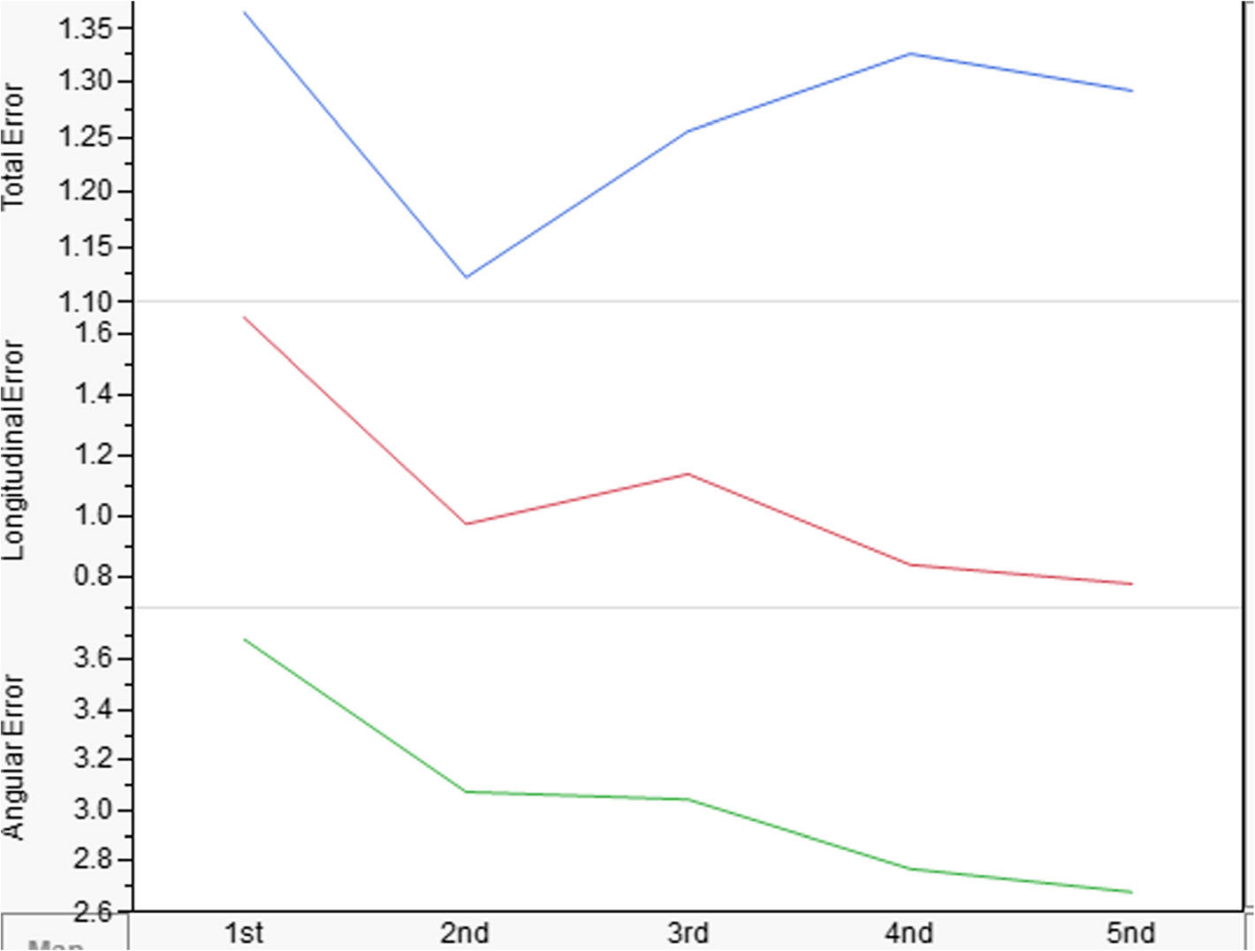
Learning curve plateaus of training process

**Fig. 3:**
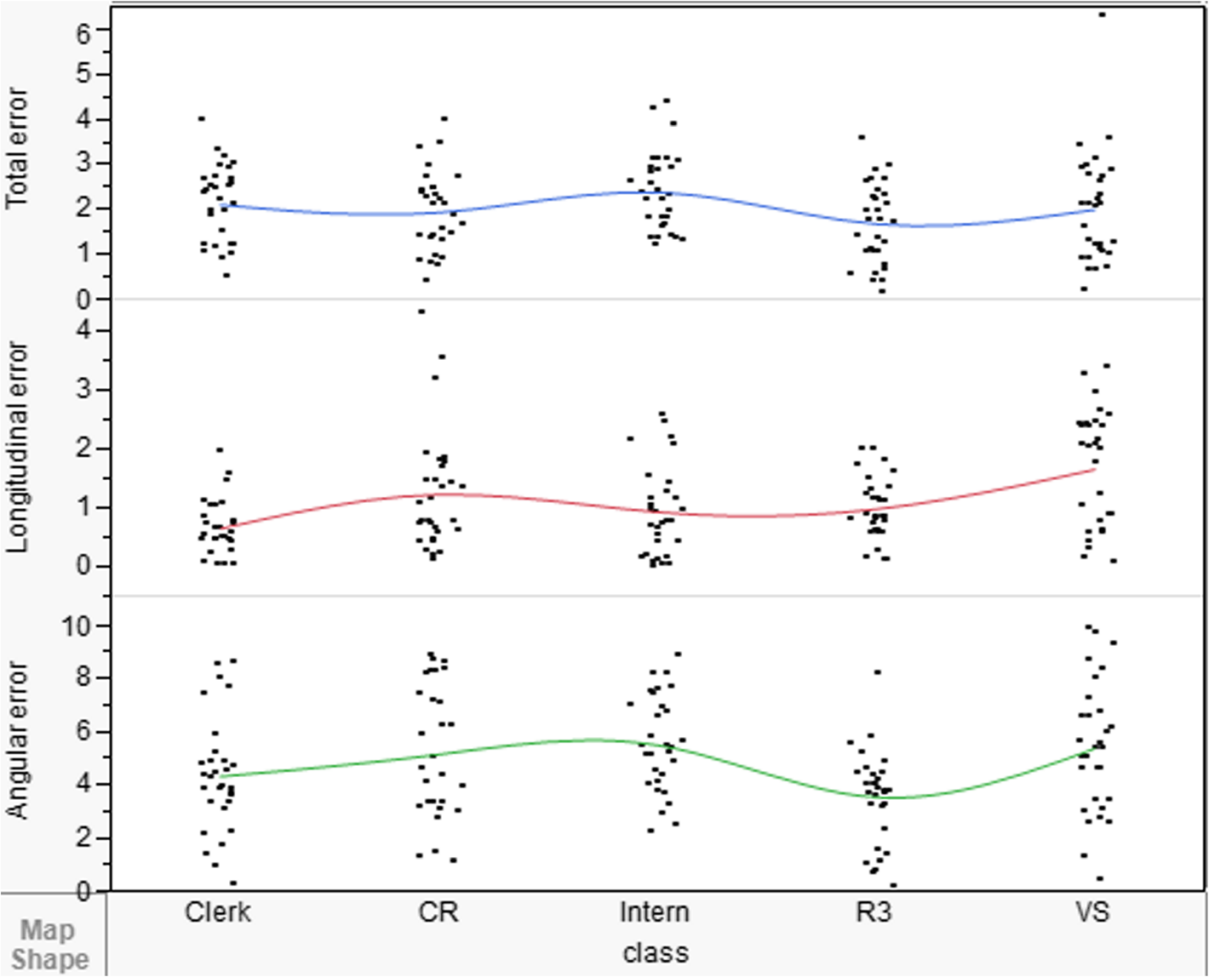
Training before the drilling test

Sun et al. [9] suggested that for this system, the plateau of the learning curve can be reached after drilling 150 holes on dental models during training. Obviously, we require all participants to reach the learning curve plateau before drilling test (Fig. 2). Moreover, the results of learning curve plateau showed a stable performance (Fig. 3), this study will commence drilling test by different experience of dentists.

### Power calculation

Chen et al. [14] reported that the total, longitudinal, and angular deviation values of Aq Navi systems were 1.07 ± 0.48 mm, 1.35 ± 0.55 mm and 4.45 ± 1.97 degrees, respectively, the minimum required sample size of 9, 12, 15 drilling holes according to total, longitudinal, and angular deviations, respectively, was separately calculated using a statistical software (Stata Statistical Software: College Station, TX: Stata Corp LP). for One-Way ANOVA F test with 80% of study power and significant level (α) of 0.05.

### Drilling test

Subsequently, using 5 pairs of dental models, each participant drilled 5 implant holes at 6 partially edentulous positions (11, 17, 26, 31, 36, and 47). In total, each participant conducted 30 drilling tests. The accuracies were evaluated by comparing the positions of actually drilled holes and those of the holes planned using software before surgeries. The following steps were followed to conduct this comparison.

#### Step 1. Preparation of the tooth model and conducting CT scans

**Fig. 4:**
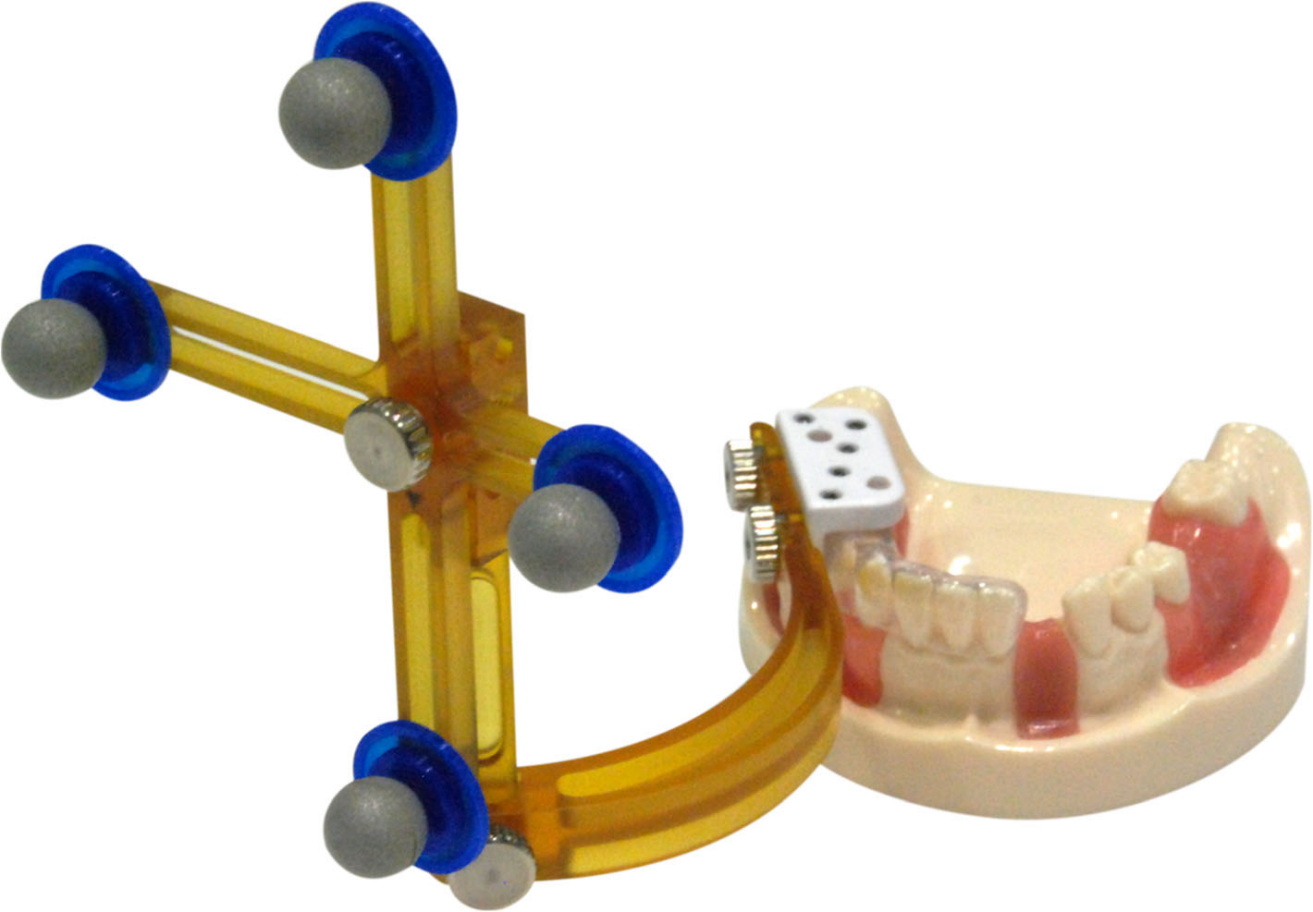
Tracking targets in Nissin dental models might occasionally block the surgeon’s view. (Figure was provided by Taiwan Implant Technology Company, Ltd.)

The Nissin dental models (Nissin Dental Products) illustrated in Fig. 4 were used to obtain the desired edentulous conditions (maxillary edentulous positions: 11, 17, and 26; mandibular edentulous positions: 31, 36, and 47). The models were replicated using polyvinylsiloxane impression materials (Aquasil Monophase, Dentsply). The impression materials were poured into type III dental stone (ADA Specification No. 25) to produce 25 sets of dental models (each set contained 1 model for the maxilla and 1 for the mandible, hereinafter referred to as the dental model). All the models were made 3 days before the tests and were stored in a moisture-proof plastic container. In the pilot study, a reference scanner (Activity 880) and CBCT (cone beam computed tomography) (voxel size: 0.15 × 0.15 × 0.15 mm, AZ3000CT) were used to confirm that all the dental models had the same tracking location as the Nissin dental model. STL (Stereolithography) files were produced using the reference scanner, and each dental model was used to drill holes at 3 edentulous positions (differences between actual drilling and preoperatively planned positions were indicated by markers); additionally, a patient tracking module was created. CBCT scans were performed on the model before and after drilling, and DICOM (Digital Imaging and Communications in Medicine) files were acquired to analyze the differences in implant positions.

#### Step 2. Preoperative planning

Before the tests, imaging data including the model number and DICOM files were first imported into the implant preoperative planning software program (SmilePlan, TITC Ltd) in the AqNavi system for surgical planning. This system was active; it utilized an infrared tracking system comprising emitters, a camera, and a tracking data processor to determine the location and orientation of the dental handpiece relative to the patient. The first planned implant was placed in the desired surgical position in the anterior edentulous area; on the other side, the second planned implant had a diameter of 3.5 mm and a length of 8 mm; it was placed in the desired surgical position in the posterior edentulous area. The original data set was established and was compared with data obtained from tests performed by different participants.

#### Step 3. Preoperative correction and drilling tests

To simulate real-time navigation surgery, the participant was required to perform calibration using the iterative closest point (ICP) method. In this method, the positions of barium sulfate beads on the calibration template and the beads close to dental implants in the CBCT image space were aligned to the planned implants in the system. If this calibration step is repeatable and reliable, it is possible to correct the transformation matrix for the CBCT image coordinate system in the image space and to obtain accurate ramifications of the surgery coordinate system during surgery. After completing planning, the surgeon conducted a simulated dental implant surgery based on the position, angle, and depth provided by the images of the navigation system.

#### Step 4. Image instructions

**Fig. 5:**
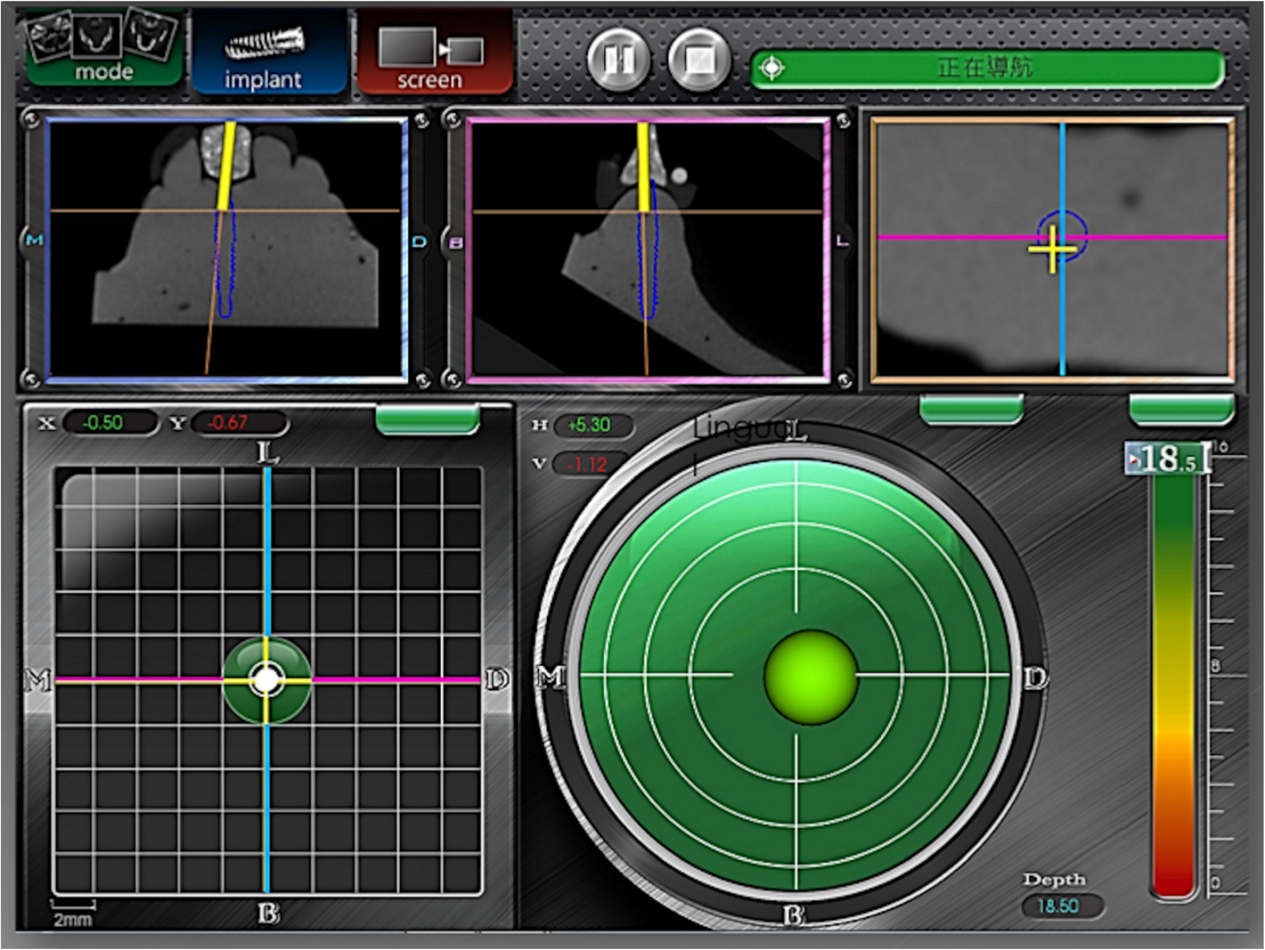
Navigation monitor showing real-time implant drilling. Three-dimensional (3D) image monitor helps surgeon to correct the error immediately

Using the guidance provided by the surgical navigation system, the participant drilled holes with a 2.0-mm guiding drill, followed by a 3.5-mm drill, until the expected depth was reached. As illustrated in Fig. 5, for the position indicator, a real-time traceable red cross was used to indicate the actual position, and the center of the screen was designed as the planned correct position. To make it easier for dentists to accurately read and interpret the screen, coordinates were designed to accommodate both buccal–lingual and mesial–distal directions. For the angle indicator, a real-time traceable ball was used to indicate the current deviated angle, and the center of the screen indicated the planned correct angle. For the depth indicator, a bar was used to indicate the distance to the correct position of apex. A pane in the upper right corner of the depth indicator indicated whether the depth of the drill exceeded the correct depth.

#### Step 5. Postoperative measurement

After completion of the experiment, CT (Computed Tomography) back scanning was performed on the dental models. The actual drilling plan was constructed using the SmilePlan software program, and the imaging data from the second CT scan were imported into the SmilePlan software program.

#### Step 6. Definition of preoperative and postoperative coincidence methods

First, the ceramic beads on the patient tracking modules were measured to obtain the bead positions with Smile Plan software; results were written to separate files. These 2 spatial coordinate files were imported using a SolidWorks combination file. During positioning, the oral positioning plates of the 2 spatial coordinate files were combined according to the point (the point was the in-built oral positioning point of the SmilePlan software), line (the second longest length of the triangle connected by the outermost 3 points of oral positioning points), and surface (the triangle connected by the 3 outermost points of the oral positioning points).

**Fig. 6:**
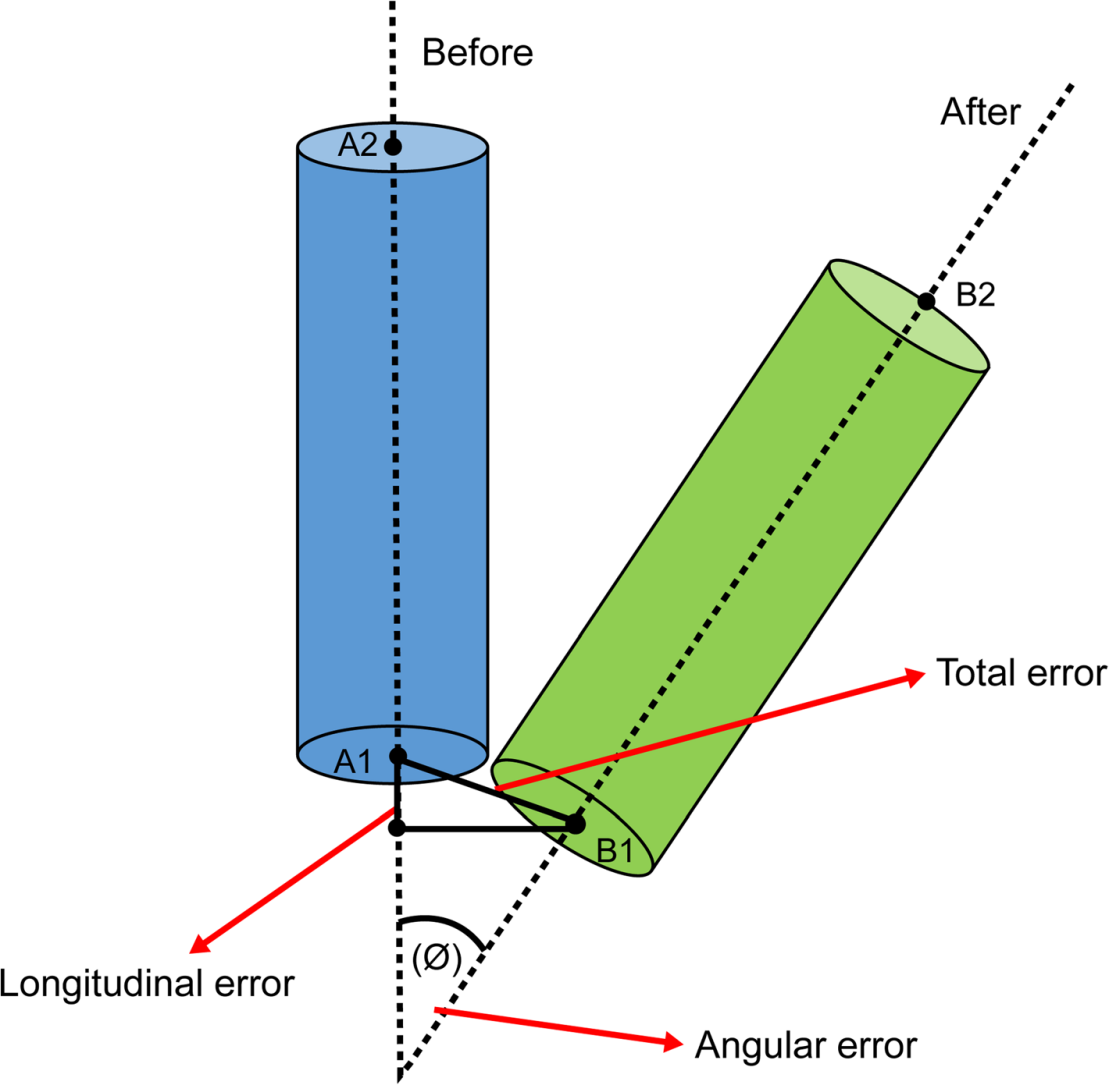
Measurement of the total error (mm), longitudinal error (mm), and angular error (°) before and after the tests

The function of creating a new axis in SolidWorks software was used to establish the coordinate axis on the implant axis in the control group. As presented in Fig. 6, the measurement function was used to directly measure the endpoints of the implant axis in the experimental and control groups. Three sets of values, namely dx, dy, and dz., were obtained, and the square roots of dx and dy were used to obtain the total error of the experiment; dz. was used to obtain the “longitudinal” error. The product of spatial vectors used to obtain the angle between vector A ($\overrightarrow{A}$) and vector B ($\overrightarrow{B}$) was the angular error (∅).^15^

### Statistical methods

The mean and standard deviation in 5 different experience levels of dentists and 6 regions among the 3 errors (total, longitudinal, and angular errors) were calculated through one-way analysis of variance (ANOVA), followed by the Tukey–Kramer multiple comparison test. One-way repeated-measures ANOVA was used to analyze differences among the five dentists in terms of trueness measurement for the error deviations (total, longitudinal and angular). Moreover, the intraclass correlation coefficient (ICC) was calculated to verify reproducibility of the superimposition in each error deviation. Analyses were performed using the JMP statistical software program (JMP, SAS Institute, Inc. 2003). Power calculated using a statistical software (Stata Statistical Software: College Station, TX: Stata Corp LP).

## Results

### Comparison of Total error, longitudinal error, and angular error among five dentists

Total error, longitudinal and angular error had satisfied reproducibility. ICCs for five dentists were separately 0.58 (95% confidence interval [CI] 0.206, 0.949), 0.86 (95% confidence interval [CI] 0.598, 0.983) and 0.79 (95% confidence interval [CI] 0.402, 0.974). By using one-way repeated-measures ANOVA, differences in trueness measurement for five dentists. The longitudinal and angular error showed significant differences (*P* = 0.0000 and *P* = 0.0453).

### Findings of the 5 participants who used the implant navigation system

As shown in Table 1, the operating time required by the VS relative to other experience level classifications was short (*P* < .05). The boreholes created using the AqNavi System showed average deviations of total, longitudinal, and angular errors of 2.00 ± 1.24 mm, 1.65 ± 0.98 mm, 5.38 ± 2.45°, respectively, in the VS test. In the CR test, these values were 1.92 ± 0.90 mm, 1.24 ± 0.99 mm, and 5.12 ± 2.48°. In the R3 test, the corresponding values were 1.66 ± 0.89 mm, 0.98 ± 0.53 mm, and 3.48 ± 1.76°. In the DI test, these values were 2.40 ± 0.87 mm, 0.92 ± 0.76 mm, and 5.53 ± 1.81°. In the DC test, the average deviation values, which provided information on the accuracy of implantation, were 2.10 ± 0.85 mm, 0.64 ± 0.46 mm, and 4.31 ± 2.13°. A comparison of the tests performed by different dentists demonstrated significant differences in the longitudinal error (*P* < .0001) and in the angular error (*P* = .0011). However, a comparison of the tests performed by 5 dentists revealed that the differences in total error were not statistically significant. According to one-way ANOVA and Tukey–Kramer HSD tests, significant differences were observed in the longitudinal error and angular error among the tests. By using dental navigation system, it can shorten the difference cause by experience. Especially, the deviation of total error was not statistically significant.

**Table 1:** The deviation of total error, longitudinal error, and angular error in the 5 drilling tests

Grade	VS	CR	R3	DI	DC	*P*-value^a^	Multiple comparison^b^
	Mean ± SD	Mean ± SD	Mean ± SD	Mean ± SD	Mean ± SD		
Total error (mm)	2.00 ± 1.24	1.92 ± 0.90	1.66 ± 0.89	2.40 ± 0.87	2.10 ± 0.85	0.054	
Longitudinal error (mm)	1.65 ± 0.98	1.24 ± 0.99	0.98 ± 0.53	0.92 ± 0.76	0.64 ± 0.46	< 0.0001	VS > DC VS > DI VS > R3 CR > DC
Angular error (degrees)	5.38 ± 2.45	5.12 ± 2.48	3.48 ± 1.76	5.53 ± 1.81	4.31 ± 2.13	0.0011	DI > R3 VS > R3 CR > R3
Elapsed time (secs per drill)	112 ± 13.13	136 ± 17.47	144 ± 5.29	170 ± 5.59	180 ± 4.49	0.0029	

^a^One-way ANOVA (*P* < .05)

^b^Tukey-Kramer HSD

CR: Chief resident; DC: Dental clerk; DI: Dental intern; R3: Third year resident; VS: Visiting staff

### Deviation of Total error, longitudinal error, and angular error by the maxilla and mandible

The total error of drilling tests showed no significant differences in the maxilla (*P* = .32, Table 2). However, the deviation of the total error in the mandible was higher in the DI test than in the CR test (*P* = .035). The longitudinal error in the maxilla showed significant differences between VS and CR tests (*P* = .03). The longitudinal error in the mandible showed significant differences between dentists (*P* < .05). The angular error in the maxilla showed no significant difference (*P* = .30); however, CR had more deviations in the mandible than DC (*P* = .01).

**Table 2:** The deviation of total error, longitudinal error, and angular error between maxilla and mandible

	Maxilla (Mean ± SD)					*P*-value^b^	Mandible (Mean ± SD)					*P*-value^b^
	VS	CR	R3	DI	DC	MC^c^	VS	CR	R3	DI	DC	MC^c^
Total Error (mm)	2.15 ± 1.44	2.48 ± 0.77	2.26 ± 0.95	2.52 ± 0.87	1.82 ± 0.82	0.3193	1.85 ± 1.02	1.36 ± 0.64	1.95 ± 0.74	2.28 ± 0.87	1.52 ± 0.92	0.0351
												DI > CR^a^
Longitudinal Error (mm)	1.31 ± 0.91	0.77 ± 0.52	0.54 ± 0.39	0.83 ± 0.83	0.98 ± 0.46	0.0309	2.00 ± 0.96	1.70 ± 1.14	0.75 ± 0.52	1.01 ± 0.69	0.97 ± 0.61	0.0002
						VS > CR^a^						VS > R3^a^ VS > DC^a^ VS > DI^a^ CR > R3^a^
Angular Error (degrees)	6.47 ± 3.37	4.96 ± 1.98	5.79 ± 2.26	6.36 ± 2.86	4.99 ± 1.56	0.2978	6.34 ± 3.28	8.12 ± 6.25	5.00 ± 3.76	7.03 ± 2.39	3.15 ± 2.23	0.0088
												CR > DC^a^

^a^One-way ANOVA (*P* < .05)

^b^Tukey-Kramer HSD

^c^MC: Multiple comparison

CR: Chief resident; DC: Dental clerk; DI: Dental intern; R3: Third year resident; VS: Visiting staff

## Discussion

In recent years, learning theories have been employed to verify whether surgeon experience improves surgical outcomes and reduces medical resource consumption. Dental implant surgical experience has long been regarded as a crucial factor for the success of dental implant surgery. The goals of dental implant navigation systems are to increase accuracy in dental implant surgery and to minimize the occurrence of unnecessary medical disputes and negligence due to surgeon inexperience. Gasparini et al. [15] argued that mastery over the use of dental implant navigation systems and accuracy in their use can be achieved through practice. Sun et al. [10] conducted that reaching the plateau of a learning curve represents mastery over dental implant navigation system use. Breaux et al. [11] demonstrated that learning curve be established is important as an increasing number of surgeons seek to add surgery to their practice. Block et al. [16] suggested that there is a learning curve to achieve proficiency by using dental navigation system. The relationship between surgical experience and the accuracy of implant installation through this dental navigation systems could be negligible. The total error showed no significant differences between the different experience levels classified in the present study; this was consistent with the results of Block et al. [16] Although the results revealed that participants with less experience exhibited higher performance (ie, longitudinal error and angular error) than those with more experience, the use of dental implant navigation systems can be useful for both inexperienced and highly experienced dentists. The navigation system can be used to decrease the operating times of dentists with advanced implant skills, and relatively inexperienced surgeons can follow the guide to ensure accuracy in implant installation. Among participants with considerable experience of implant surgery, their experience determines the final outcomes of surgery to a greater extent than does the guidance provided by implant navigation systems. Agachan et al. [17] demonstrated the impact of surgeon experience on the complication rate, and these studies have shown a significant decrease in the complication rate as experience is gained. Agha et al. [18] reported that new technique not only proved to be safe, but also resulted in a shortened total operating-time after a learning curve. Cutting-edge equipment use may assist surgeons in achieving favorable surgical outcomes.

Geng et al. [19] who evaluated the clinical outcomes of implants placed using different types of surgical guide. They found that surgical guides can simplify surgery and aid in accuracy of implant placement. These findings of Lee et al. [20] who confirms that the use of surgical guide is an effective way to improve the accuracy of implant placement. Gillot et al. [21] indicated that dental implant surgery in the posterior area is relatively difficult because of soft tissue obstruction and the short interdental space, both of which significantly affect the ability of dentists to properly place the surgical drill. In the present study, no significant differences were observed between maxillary and mandibular surgical outcomes achieved using a real-time dental implant navigation system; in particular, the system could eliminate interference from surgical guides.

The implant navigation system features several advantages; however, some drawbacks of the system must be overcome. In the AqNavi System, the tracking target is positioned in an affected site of the patient or on the surgical instrument. Because the width of the mouth is small, the tracking target occasionally blocks the surgeon’s view and gestures, thereby rendering surgery more difficult and complex because of the coordination required between the surgeon and system. In addition, the operator must simultaneously view the image guide on the screen and the actual position of the handpiece; this can cause hand-eye coordination problems due to the different orientations of the surgical site and image. Similarly, the orientations displayed on the screen are consistent with the common buccal–lingual and mesial–distal directions used by dentists to effectively reduce difficulties related to eye-hand coordination during dental implant navigation system use. Furthermore, for convenient use of the dynamic surgical navigation system and tracking guide, additional preparation steps are required before performing surgery; such steps are likely to increase surgery duration.

All the 5 dentists with different experience levels had completed the dental implant navigation system operation training and their learning curve had reached a plateau before they were allowed to participate in the experiment. The experiment results revealed that junior dentists had superior performance in terms of accuracy than senior dentists. This may be explained by the following two factors. First, instant feedback is required for effective learning. Ericsson and Pool [22] noted that experienced surgeons are able to reduce patients’ subsequent complications, especially the recurrence rate in patients with cancers. For surgeons, patients’ feedback is instant; vascular rupture and tissue damage are all immediately shown when the operation is unsatisfactory, and these errors can be immediately reviewed and corrected during the surgery. In this study, the effective feedback principle of the dental implant navigation system enabled dentists to promptly complete surgeries through system guidance. This can substantially shorten the time required for a dentist to become an expert and is also good news for patients because they can be prevented from being surgical failure cases due to dentists’ lack of experience. To ensure surgery quality, the training for dental implant surgery should be based on teamwork lead by experienced dentists; the involved dentists can therefore learn from each other. The worst training is considering the surgical process as the production line, where each dentist learns the surgery alone without cooperation.

Second, dentists’ performance may also be influenced by psychological factors. The American psychologist Duckworth [23] argued that grit influences how a person perform a task excellently. She noted that a person must achieve success through two models: talent × effort = skill and skill × effort = achievement. Specifically, people should first set a goal and put all their effort to achieve the goal. After seeking other people’s opinions and obtaining feedback, they can correct what has been done to make improvement. Accordingly, young dentists with their own talents devote efforts to acquire surgical skills; through experience accumulation, their skills can be improved and eventually accomplish remarkable achievements. Therefore, having the assistance of technology in providing guidance and feedback, young dentists will fully devote themselves into the learning and accumulate experience, thereby shortening the process of achieving success. The aforementioned two factors can help justify the results of this study.

This study examined whether a surgeon’s experience of dental implant surgery affects surgical accuracy when operating an implant navigation system. During the experiment, all major errors that could not be overlooked were controlled for in advance (eg, molding, X-ray imaging, software, and camera errors). Adhering to the standard operating procedures of the navigation system can improve the accuracy of implant placement. This study had 2 limitations: 1) the 5 participants were selected from prosthodontic department and they had little or no experience of dental installation and 2) factors such as intraoral elasticity and moisture could not be simulated.

## Conclusions

This study pre-controls all relevant factors that influence the deviation of errors. In order to reduce the selection bias, the participants must have the same educational background and training procedures. To reach the learning curve plateau of using dental navigation system before the drilling test. The results of drilling test were analyzed by statistical power, intraclass correlation coefficient, one-way repeated ANOVA and ANOVA Tukey-HSD to ensure the representativeness and consistency. Dentists can compensate for differences in experience of dental implantation through the systems. With less dental implantation experience of dentist has a longer time to operate this system. The results demonstrated that no significant differences in the total error deviation between different experience of dentists. Using the dental navigation system, dental students can be introduced to dental implant surgery earlier than what was possible in the past. It can be shortened 10,000 h of training time through this system. The implant navigation system assists the dentist in the ability to accurately insert the dental implant into the correct position without being affected by his/her own experience of implant surgery. Through the assistance of technology, we can get a lot of resources and shorten the learning process, instead of accumulating experience through clinical surgery in exchange for success.

## Data Availability

The datasets used and/or analysed during the current study are available from the corresponding author on reasonable request.

## Abbreviations

CAD-CAM: Computer Aided Design/Manufacturing
CBCT: Cone Beam Computed Tomography
CT: Computed Tomography
DICOM: Digital Imaging and Communications in Medicine
STL: STereoLithography
TITC: Taiwan Implant Technology Company, Ltd.
